# Identification of an immuno-dominant protein from *Klebsiella pneumoniae* strains causing pyogenic liver abscess: implication in serodiagnosis

**DOI:** 10.1186/s12866-014-0321-4

**Published:** 2014-12-21

**Authors:** Tzu-Lung Lin, Yi-Ping Chuang, Yu-Tsung Huang, Pei-Fang Hsieh, Yi-Tsung Lin, Jin-Town Wang

**Affiliations:** Department of Microbiology, National Taiwan University College of Medicine, 1, Sec 1, Jen-Ai Rd., Taipei, Taiwan; Department of Microbiology and Immunology, National Defense Medical Center, Taipei, Taiwan; Department of Internal Medicine, Far Eastern Memorial Hospital, New Taipei City, Taiwan; Division of Infectious Diseases, Department of Medicine, Taipei Veterans General Hospital, Taipei, Taiwan; School of Medicine, National Yang-Ming University, Taipei, Taiwan; Department of Internal Medicine, National Taiwan University Hospital, Taipei, Taiwan

**Keywords:** *Klebsiella pneumoniae*, Pyogenic liver abscess, Antigen, Serodiagnosis

## Abstract

**Background:**

*Klebsiella pneumoniae* has emerged worldwide as a cause of pyogenic liver abscess (PLA) often complicated by meningitis and endophthalmitis. Early detection of this infectious disease will improve its clinical outcome. Therefore, we tried to isolate immunodominant proteins secreted by *K. pneumoniae* strains causing PLA.

**Results:**

The secreted proteins of the NTUH-K2044 strain were separated by two-dimensional electrophoresis and then immunoblotted using convalescent sera from patients with *K. pneumoniae* PLA. A ~30-kDa immunodominant protein was then identified. Liquid chromatography-tandem mass spectrometry (LC-MS/MS) revealed an open reading frame (KP1_p307) located on the pK2044 plasmid and bioinformatic analysis identified this protein as a signal peptide of unknown function. The KP1_p307 gene was more prevalent in PLA strains and capsular type K1/K2 strains, but disruption of this gene in NTUH-K2044 strain did not decrease virulence in mice. Ten of fourteen (71%) sera from patients with *K. pneumoniae* PLA were immunoreactive with the recombinant KP1_p307 protein. Seroconversion demonstrated by a rise in serum titer in serial serum samples confirmed that antibodies against the KP1_p307 protein were elicited after infection.

**Conclusions:**

The KP1_p307 protein could be used as an antigen for early serodiagnosis of *K. pneumoniae* PLA, particularly in K1/K2 PLA strains.

## Background

*Klebsiella pneumoniae* is a common Gram-negative enteric bacterium that causes hospital-acquired urinary tract infection, septicemia, and pneumonia as well as community-acquired pneumonia. Recently, community-acquired pyogenic liver abscess (PLA) caused by *K. pneumoniae* complicated with metastatic meningitis and endophthalmitis has emerged globally especially in Asia [1–13]. Based on analysis of data from the National Health Insurance Database in Taiwan, cases of PLA per year have increased from 1,950 in 1997 to 3,083 in 2008 [14,15]. Among the 506 case-patients with PLA admitted to National Taiwan University Hospital (NTUH) from 2000 through 2004, 358 case-patients had positive culture results, and 286 (79.9%) of these 358 case-patients had positive culture results showing *K. pneumoniae* infection [15]. In a nationwide prospective study of PLA in Korea, *K. pneumoniae* was the most common etiologic organism (78.2%) [2]. *K. pneumoniae* was also reported as the most common etiological organism of PLA in New York, Hong Kong, Singapore, and Australia [2–11]. The mortality rates were 10% among those with *K. pneumoniae*-caused PLA and 30–40% among those with metastatic meningitis. The survivors of meningitis usually had severe neurological sequelae and *K. pneumoniae* endophthalmitis usually resulting in blindness of the affected eyes.

A previous study identified the predictors of septic metastatic infection and mortality among patients with *K. pneumoniae* liver abscess [16]. Most of the severe complications occurred within the first 3 days after hospital admission. Percutaneous drainage of primary liver abscess caused by *K. pneumoniae* lowered rates of mortality, metastatic infection, and complications. A previous study also observed that timing of appropriate antimicrobial therapy is a major determinant of survival and neurological outcome for patients with *K. pneumoniae* meningitis [17]. Therefore, rapid diagnosis and proper treatment for this invasive disease are required.

When a bacterial infection is suspected because of symptoms (such as fever) and increased white blood cell counts, the standard procedure in hospitals is to isolate and identify the bacterium. Identification of *K. pneumoniae* using automated or manual biochemical tests requires at least 24 to 48 hours. However, identification through molecular or immunologic techniques of bacterial nucleic acid and antigen detection can accelerate the diagnosis of bacterial infectious diseases. A real-time PCR assay with specific primers and hybridization probes targeting 16S rRNA gene was developed for the direct detection of *K. pneumoniae* from positive blood culture bottles [18]. But this method still requires culture enrichment.

In this study, we used a proteomic strategy to identify an immunodominant protein in *K. pneumoniae* strains causing PLA and evaluated the sensitivity and specificity of an immunoblot method for detecting antibodies to this antigen in patient sera.

## Methods

### Ethics statement

All the clinical bacterial strains used in this study were provided by the strain collection of National Taiwan University Hospital (NTUH) in Taiwan. The Ethics Committee approved that no formal ethical approval was needed to use these clinically obtained materials, because the strains were remnant from patient samples, and the data were analyzed anonymously. The collection and study of serum samples in this study was approved by the Institutional Review Board of NTUH and written informed consent was obtained from each participant (approval number: 200904046R). All animal experiments followed the guidelines in the Handbook of Laboratory Animal Care of the National Laboratory Animal Breeding and Research Center, National Science Council of Taiwan, and were approved by the Institutional Animal Care and Use Committee of the National Taiwan University College of Medicine (approval number: 20130005).

### Bacterial strains and culture conditions

The 74 *K. pneumoniae* clinical isolates used in this study were collected from NTUH from 1997 to 2003 [19]. Forty-two were PLA strains isolated from the blood of patients with PLA with or without meningitis or endophthalmitis complications, and 32 were non-tissue-invasive strains isolated from patients with sepsis but not PLA or other metastatic infections in any tissue. *K. pneumoniae* and *Escherichia coli* were cultured in LB medium at 37°C supplemented with appropriate antibiotics, including 50 μg/ml kanamycin and 100 μg/ml ampicillin.

### Serum samples

The serum samples from fourteen patients with *K. pneumoniae* PLA and three patients with non-*K. pneumoniae* liver abscess were obtained from NTUH in 2008 ~ 2010. The dates of admission and sera collection were recorded. The diagnosis of liver abscess was confirmed by abdominal sonography or computed tomography, as well as isolation and identification of the pathogens from blood or abscess aspiration. The serum samples from twelve patients with *K. pneumoniae* infections (other than PLA) were obtained from Taipei Veterans General Hospital (VGH) in 2012. The diagnosis was based on clinical presentations and history.

### Purification of secreted proteins

NTUH-K2044 strain was cultured overnight and pelleted by centrifugation. The culture supernatant was collected and 0.45-μm filtered. The secreted proteins were precipitated by adding trichloroacetic acid to a final 20% concentration and incubating on ice for 30 minutes, collected after centrifugation, washed several times with cold acetone, air-dried, dissolved in 1.5 M Tris-Cl (pH 8.8), and assayed to determine protein concentration using the Bradford method.

### Two-dimensional gel electrophoresis and immunoblotting

The proteins (2–3 mg) were separated by two-dimensional electrophoresis. Briefly, protein extract in sample buffer (8 M urea, 2% Pharmalyte pH 3–10, 60 mM DTT, 4% CHAPS, bromophenol blue) was first separated on IPG strips (Immobiline DryStrip pH 3–10), then on 12% sodium dodecyl sulfate (SDS)-polyacrylamide gels (PAGE; 18 cm × 20 cm), and the separated proteins were transferred onto a poly(vinylidene) fluoride (PVDF) membrane (Millipore, Bedford, MA). The membrane was incubated with blocking buffer (5% skimmed milk in PBS with 0.5% Tween 20) for 1 h at room temperature, primary antibody (sera from patients or healthy subjects or anti-GST antibody or anti-KP1_p307 antibody diluted in blocking buffer), secondary antibody (horseradish peroxidase [HRP]-conjugated goat anti-human IgG or HRP-conjugated goat anti-rabbit IgG [Chemicon, Temecula, CA]), and then enhanced chemiluminescence (ECL) reagent.

### Liquid chromatography-tandem mass spectrometry (LC-MS/MS) analysis

The protein spots on the Coomassie blue stained two-dimensional gel were excised and digested by trypsin. The peptide mixture was analyzed using a nanoAcquity system (Waters, Milford, MA) connected to an LTQ-Orbitrap Elite hybrid mass spectrometer (Thermo Fisher Scientific, Bremen, Germany) equipped with a nanospray interface (Proxeon, Odense, Denmark).

### Expression and purification of recombinant KP1_p307 protein

KP1_p307 gene was amplified by PCR using primers KPP307-074F (5′-CCGTCGCCGGCGATAAATC-3′) and KPP307-916R (5′-CGTAGATATCAGCGTTACCAAAG-3′) and then cloned into a pGEM-T easy plasmid. The KP1_p307 was sub-cloned into the Klenow fragment blunting-EcoRI site of a pGEX-4T-1 plasmid. The resulting plasmid was transformed into *E. coli* DH10B. KP1_p307 gene was amplified by PCR using primers KPP307-076F (5′-GTCGCCGGCGATAAATCCGG-3′) and KPP307R (5′-TTATTCGTAGATATCAGCGTTACC-3′) and cloned into the blunt-end SmaI site of a pQE32 plasmid. The resulting plasmid was transformed into *E. coli* M15/pREP4. The recombinant GST-fused KP1_p307 protein and His-tagged KP1_p307 protein were expressed under 1 mM isopropyl β-D-1-thiogalactopyranoside (IPTG) induction at 37°C and purified per the manufacturer’s instructions. The immuno-reactivities to GST-fused KP1_p307 protein and His-tagged KP1_p307 protein were not different. However, the yield of purified His-tagged KP1_p307 protein was higher than that of purified GST-fused KP1_p307 protein because His-tagged protein (either in supernatant or in inclusion body) could be purified under denaturing condition. Therefore, we used the His-tagged KP1_p307 protein for further examination of the diagnostic application of KP1_p307 protein.

### Knockout of KP1_p307

The KP1_p307 gene and its flanking regions were amplified by PCR using primers 307KO-F (5′-GTCCTGCTGAAACATGACC-3′) and 307KO-R (5′-ACACTATCGTGTCACAGATG-3′). Nucleotides 64–902 of the KP1_p307 coding sequence were replaced by a kanamycin resistant gene. The deletion fragment was then cloned into the suicide vector pUT containing a spectinomycin resistant gene as a selective marker to distinguish between single and double crossovers for chromosomal integration [20,21]. The resulting plasmid was transferred to NTUH-K2044 by conjugation. Chromosomal deletion of the KP1_p307 mutant was confirmed by PCR with different primer pairs.

### Virulence assay in mice

Female 5-week-old BALB/cByl mice were inoculated with *K. pneumoniae* intraperitoneally as previously described [22]. Four mice were used to test the effects of each inoculum. After inoculation, the mice were observed for 30 days. The LD50 was calculated using the method established by Reed and Muench. For *in vivo* competition, the KP1_p307 mutant strain and the fully virulent p*lacZ* deletion mutant strain in the logarithmic phase were mixed at a 1:1 ratio (1 × 10^3^ colony-forming units each) in saline, and inoculated intraperitoneally into eight 5-week-old BALB/c mice as previously described [23]. The amounts of bacteria in livers and spleens one day after inoculation were determined by plating serial dilutions onto LB agar containing 1 mmol/L IPTG and 50 mg/mL X-gal. *In vivo* competition was assessed using the competitive index (the output to input ratio of the KP1_p307 mutant strain [blue colony] to the p*lacZ* deletion mutant strain [white colony]).

### Immunoblotting of recombinant KP1_p307 protein with patient sera

The purified recombinant His-tag KP1_p307 protein was separated on 12% SDS-PAGE (~5 ng per lane) and then transferred onto a membrane. Patients’ sera (1:2000 dilution) were screened simultaneously using Mini-PROTEAN II Multiscreen Apparatus (Bio-Rad, Hercules, CA). Those collected first were screened first, and immunoblotting with serial sera was performed when the first collected serum was antibody negative.

### Capsular typing by *wzc* sequencing

Capsular types of the *K. pneumoniae* strains were determined by *wzc* genotyping [24]. The sequences of CD1-VR2-CD2 region were compared with those of reference strains by NCBI-nucleotide blast and the corresponding capsular types were determined.

## Results

### Identification of immunodominant proteins in NTUH-K2044 strain

**Figure 1:**
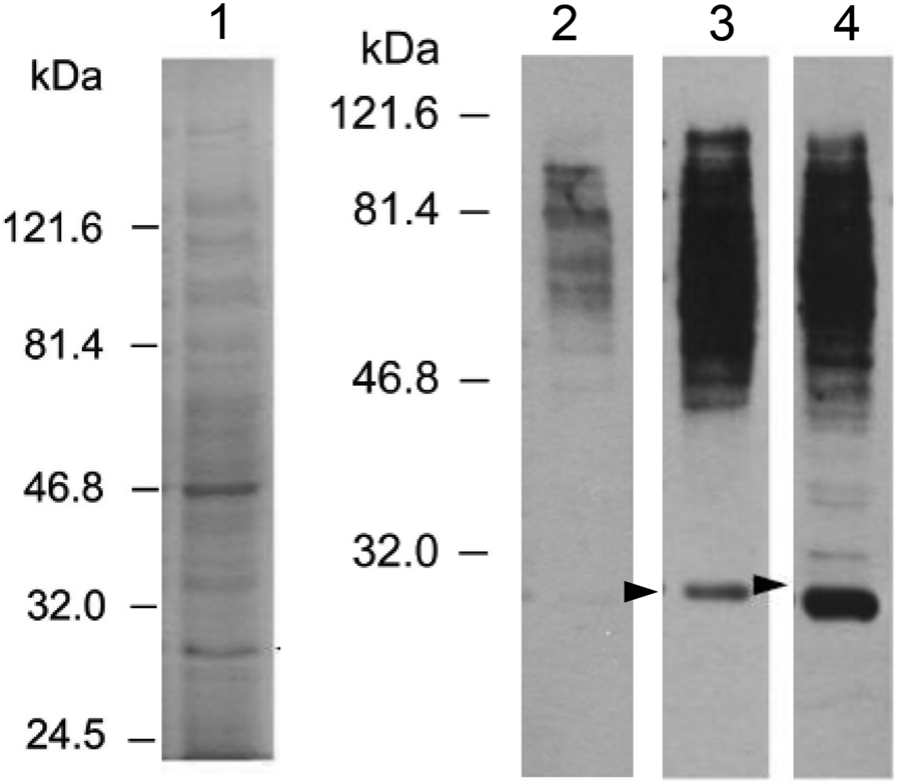
**Identification of immuno-dominant proteins in NTUH-K2044 by one dimensional-PAGE.** Lane 1, the coomassie blue staining of the secreted proteins of NTUH-K2044 strain. Lane 2, the immunoblotting with serum from healthy subject. Lane 3, the immunoblotting with serum from patient 1 with *K. pneumoniae* causing PLA. Lane 4, the immunoblotting with serum from patient 2 with *K. pneumoniae* causing PLA. The arrow heads indicate the putative protein immuno-reactive to sera from patients with PLA.

**Figure 2:**
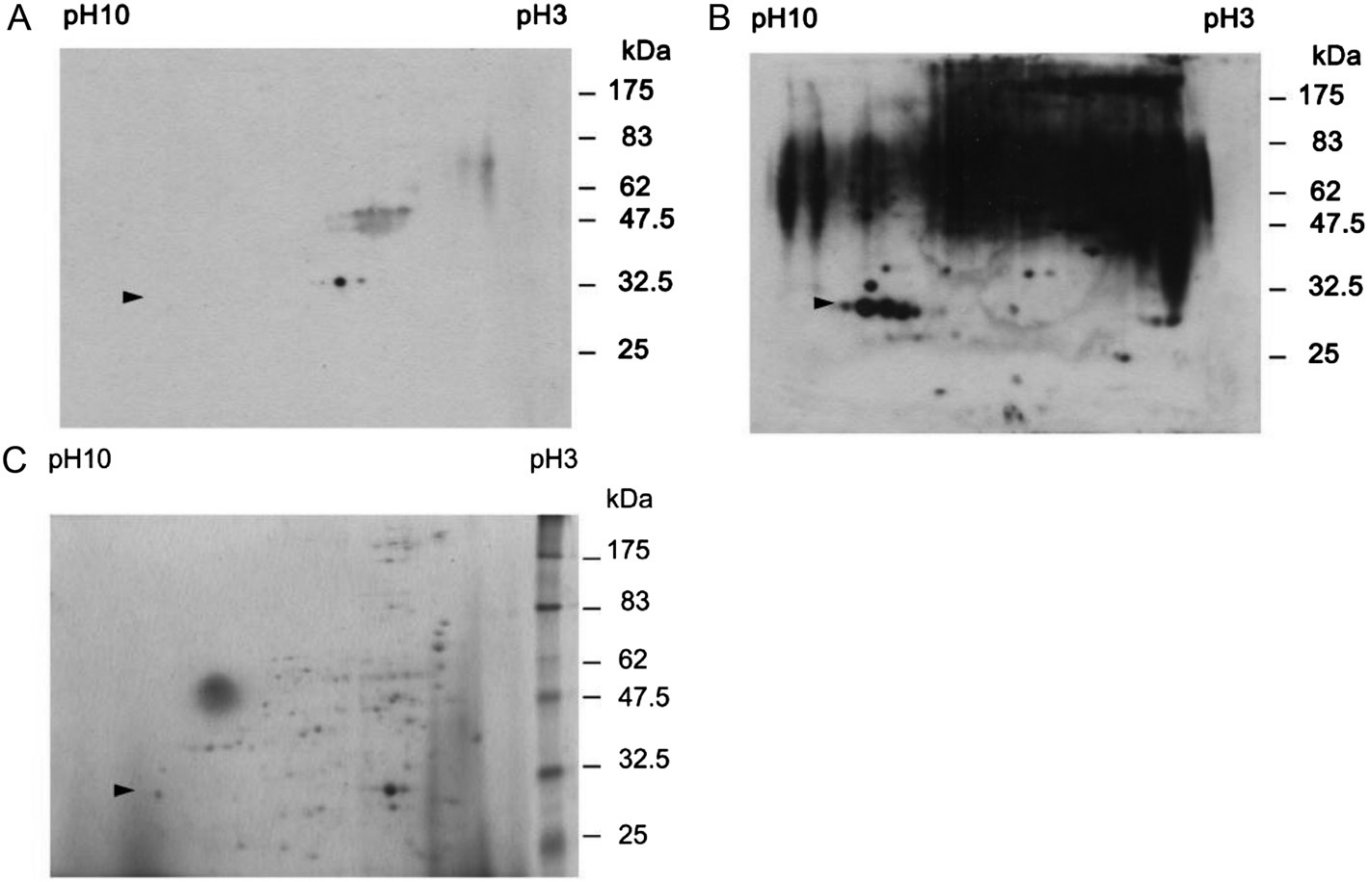
**Identification of immuno-dominant proteins in NTUH-K2044 by two dimensional-gel electrophoresis. A**, the immunoblotting with serum from healthy subject. **B**, the immunoblotting with serum from patient 2 with *K. pneumoniae* causing PLA. **C**, the coomassie blue staining of the secreted proteins of NTUH-K2044 strain. The arrow heads indicate the putative protein immuno-reactive to sera from patients with PLA.

Proteins released by bacteria to the extracellular environment are usually the antigens that elicit immune responses, have diagnostic value, and can provide protective immunity. To identify immunodominant proteins in *K. pneumoniae* NTUH-K2044 strain causing pyogenic liver abscess (PLA), the secreted proteins were isolated as described above and analyzed by immunoblotting using sera from one healthy subject and two patients in the convalescent phase of PLA caused by *K. pneumoniae* (Figure 1). The convalescent sera from patients with *K. pneumoniae* PLA were primarily used to identify immunodominant proteins secreted by *K. pneumoniae* strains causing PLA. Because sera obtained from patients in the convalescent stage were considered to be especially rich in antibodies against the infectious agent. A 30-kDa band was recognized by the sera from the two patients but not the serum from the healthy subject. Further separation by two-dimensional gel electrophoresis and immunoblotting with the above sera followed by Coomassie blue staining (Figure 2) identified a similar band (approximately 30-kDa molecular weight and isoelectric point [pI] ranging from 7 to 9) that was immunoreactive with patients’ sera and therefore a possible candidate protein for immunological diagnosis of PLA caused by *K. pneumoniae*.

### Identification of the protein immunoreactive to sera from patients with PLA

The protein spots on the two-dimensional gels were excised, digested by trypsin, and analyzed by LC-MS/MS. The obtained peptide sequences were compared with the whole genome sequences of NTUH-K2044 strain (chromosome and a large plasmid: AP006725.1 and AP006726.1). A 921-bp open reading frame (KP1_p307) located on the large plasmid pK2044 was confirmed. The sequences of peptides obtained from LC-MS/MS represented 93.1% of KP1_p307 protein sequences. The theoretical pI and molecular weight of KP1_p307 protein (i.e., 8.87 and 32,254.72-Da) matched those of the immunoreactive protein. The amino acid sequences of KP1_p307 revealed 47% (107/229) identity to a hypothetical protein of *Lactobacillus rossiae* (WP_026017099.1) and 53% (95/180) identity to a hypothetical protein of *Enterococcus dispar* (WP_016173221.1). Bioinformatic analysis revealed that this protein contained a signal peptide located at N-terminal residues 1 to 24 but had no known function.

### Confirmation of the immunoreactivity of KP1_p307 protein

**Figure 3:**
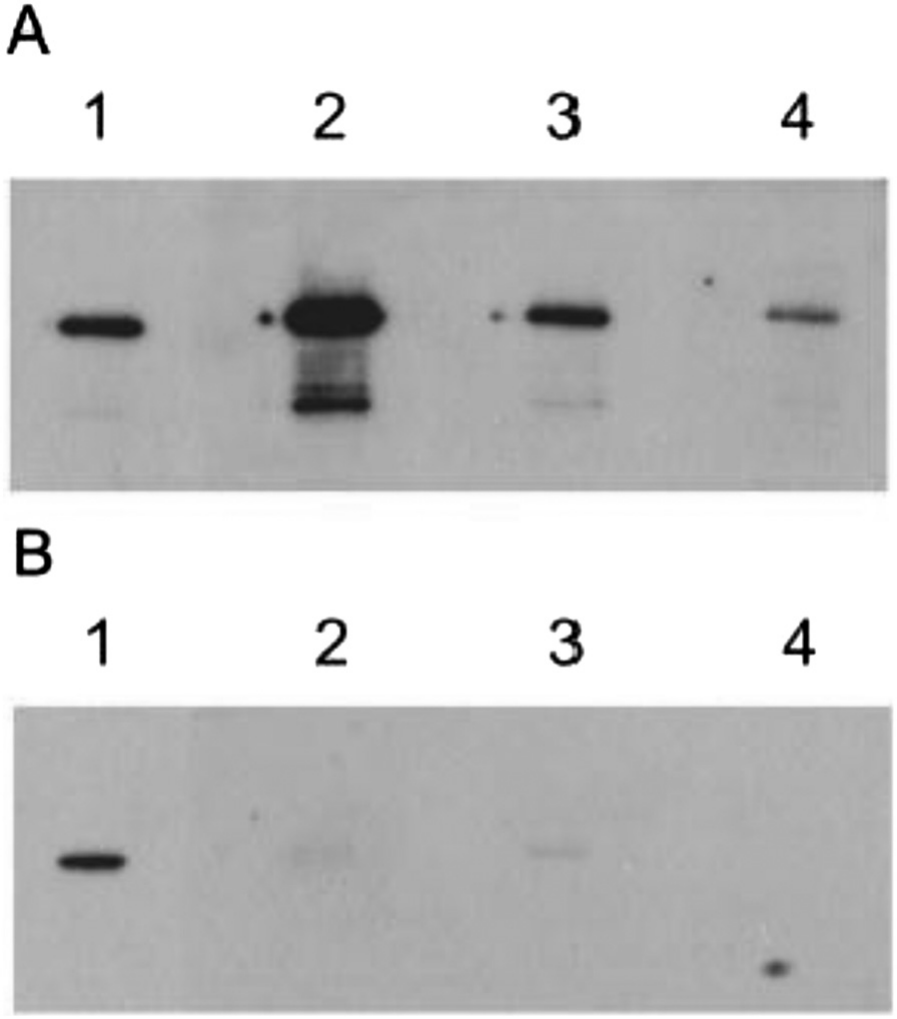
**Confirmation of the immune-reactivity of the recombinant KP1_p307 protein.** The recombinant GST-KP1_p307 protein was separated on SDS-PAGE and immunoblotted with sera from another four patients (lane 1-4 of panel **A)** or from healthy subjects (lane 2-4 of panel **B)** or anti-GST antibody (lane 1 of panel **B)**.

The recombinant GST-KP1_p307 protein was expressed and purified in *E. coli* to confirm its immunoreactivity. Sera from another four patients with PLA and three healthy subjects were used to immunoblot the recombinant GST-KP1_p307 protein. The GST-KP1_p307 protein was immunoreactive with sera from patients but not from healthy subjects (Figure 3). The immunoreactive signal of the secreted proteins disappeared when the immunoreactive serum was pre-incubated with the recombinant GST-KP1_p307 protein but not with GST protein (data not shown). These results confirmed that KP1_p307 protein was the candidate protein identified above.

### Prevalence of strains with KP1_p307

**Figure 4:**
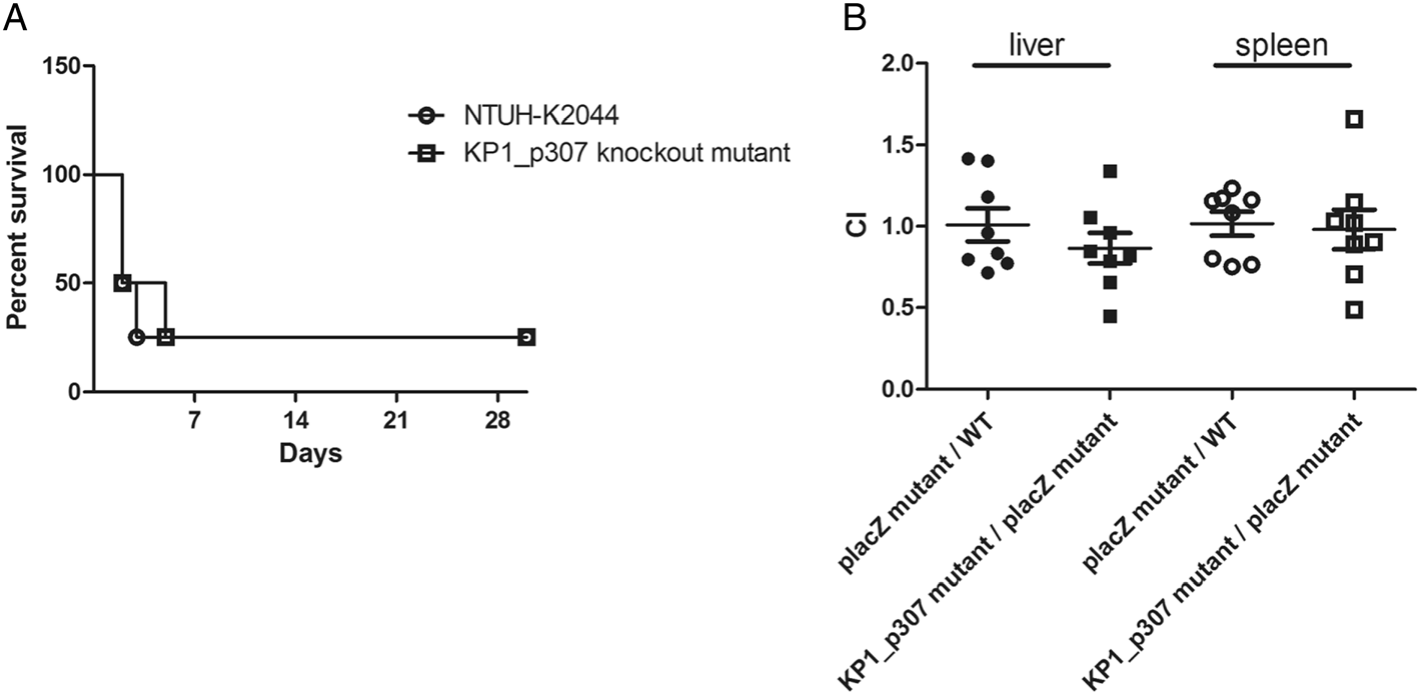
**The virulence of KP1_p307 knockout mutant. A**, The survival curve of mice with intraperitoneal infection of wild type or KP1_p307 knockout mutant. Female 5-week-old BALB/cByl mice were inoculated with 1 × 10^3^ cfu of *K. pneumoniae* wild type or KP1_p307 knockout mutant intraperitoneally (four mice each group). The survival of mice infected with wild type or KP1_p307 knockout mutant was not significantly different (*P* = 0.870, log-rank test). **B**, The *in vivo* competition between wild type and KP1_p307 mutant in liver and spleen of BALB/c mice. The wild type strain (blue colony on LB agar containing IPTG and X-gal) and the p*lacZ* deletion mutant (white colony) or KP1_p307 mutant strain (blue colony) and the p*lacZ* deletion mutant strain (white colony) were mixed at a 1:1 ratio and inoculated intraperitoneally into eight BALB/c mice. The amounts of bacteria in livers and spleens in the next day after inoculation were determined. The *in vivo* competition index (CI) was calculated. The CI of *lacZ* mutant/WT in liver is 1.009 ± 0.101 and that of KP1_p307 mutant/lacZ mutant in liver is 0.865 ± 0.094. The CI of *lacZ* mutant/WT in spleen is 1.016 ± 0.073 and that of KP1_p307 mutant/*lacZ* mutant in spleen is 0.981 ± 0.121. The *in vivo* competition of wild type and KP1_p307 mutant was not significantly different (*P* = 0.315 in liver, *P* = 0.808 in spleen, Student’s t test).

The prevalence of PLA strains and non-tissue invasive strains with KP1_p307 was determined by PCR and Western blotting using rabbit anti-KP1_p307 serum (Table 1). The results of PCR and Western blotting were consistent. The prevalence of PLA strains with KP1_p307 (39 out of 42) was significantly higher than that of non-tissue invasive strains with KP1_p307 (6 out of 32) (Chi-square analysis, *P* < 0.0001). K1 and K2 were the most prevalent capsular types causing PLA and were strongly associated with virulence. Thirty-seven of the 42 PLA strains were K1/K2, whereas 4 of the 32 non–tissue-invasive strains were found to be K1/K2. The prevalence of K1/K2 strains with KP1_p307 (39 out of 41) was significantly higher than that of non-K1/K2 strains with KP1_p307 (6 out of 33) (Chi-square analysis, *P* < 0.0001). Because this gene might be associated with bacterial virulence, a KP1_p307 knockout mutant of the NTUH-K2044 strain was generated and its virulence was tested in mice. The survival of mice intraperitoneally infected with 1 × 10^3^ cfu of wild type or KP1_p307 knockout mutant was not significantly different (Figure 4A). Knockout of KP1_p307 did not increase the lethal dose (LD50 < 1 × 10^3^ cfu) of NTUH-K2044 strain given intraperitoneally. Moreover, the *in vivo* competition between the wildtype and KP1_p307 knockout mutant strains was also not significantly affected (Figure 4B).

**Table 1:** **The prevalence of KP1_p307 among 42 PLA strains and 32 non-tissue invasive strains**

	**PLA strains (n = 42)**		**Non-tissue invasive strains (n = 32)**	
	**K1/K2**	**non-K1/K2**	**K1/K2**	**non-K1/K2**
KP1_p307 +	35	4	4	2
KP1_p307 −	2	1	0	26

The prevalence of KP1_p307: PLA strains vs. non-tissue invasive strains (*P* < 0.0001, Chi-square analysis); K1/K2 strains vs. non-K1/K2 strains (*P* < 0.0001, Chi-square analysis).

### Diagnostic application of KP1_p307 protein in *K. pneumoniae* PLA

Sera from 14 patients with *K. pneumoniae* PLA were collected to evaluate the diagnostic application of KP1_p307 protein (Table 2). Ten of the 14 (71.4%) sera were immunoreactive with the recombinant KP1_p307 protein. Because not all the PLA strains have KP1_p307, the isolates from the four patients whose sera did not react to the recombinant KP1_p307 protein were tested to determine whether they possess KP1_p307. Two out of these four strains did not have KP1_p307, and therefore could not elicit these antibodies. Moreover, sera from three patients with LA not caused by *K. pneumoniae* and sera from ten healthy subjects were not immunoreactive with the recombinant KP1_p307 protein. Therefore, presence of antibodies against the KP1_p307 protein can be implicated in *K. pneumoniae* PLA.

**Table 2:** **The immuno-reaction to KP1_p307 protein of sera from 17 patients with liver abscess**

**Patients**	**Diagnosis**	**Serum collection day (the day after admission)**						**KP1_p307 PCR**
A	*K. pneumoniae* causing PLA	**3**						ND
B		**1**						ND
C		**1**						ND
D		**26**						ND
E		**1**						ND
F		**4**						ND
G		**4**						ND
H		**1**						ND
I		3	**5**	**7**	**110**			ND
J		2	4	**10**	**14**	**25**	**39**	ND
K		2	26					+
L		1	7	22				+
M		3	7	55				−
N		1	6	14				−
O	PLA (non-*Kp*)	5	30	60				
P	PLA (non-*Kp*)	1	11					
Q	amoebic LA	1	13					

The number in boldface indicates the positive reaction to KP1_p307 protein and the number in standard indicates the negative reaction to KP1_p307 protein.

ND: not determined.

**Figure 5:**
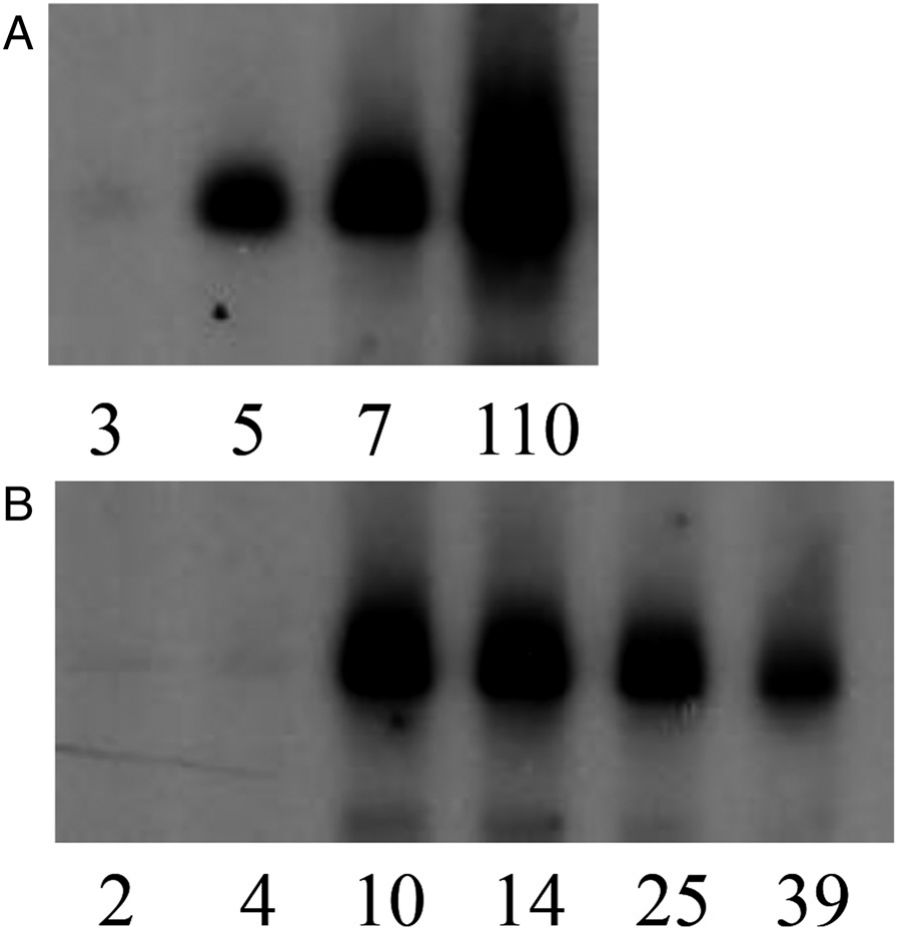
**The seroconversion to KP1_p307 in two patients with** ***K. pneumoniae*** **causing PLA.** The recombinant His-tag KP1_p307 protein was separated on SDS-PAGE and immunoblotted with serial sera collected from two patients with *K. pneumoniae* causing PLA simultaneously by using Bio-Rad Mini-PROTEAN II Multiscreen Apparatus (in panels **A** and **B**, respectively). The numbers under the panel indicate the date after admission to hospital. The seroconversion was observed in panel **A** (the 5^th^ day) and in panel **B** (the 10^th^ day).

Antibodies against the KP1_p307 could be detected in the first serum sample (collected 1–26 days after admission) from eight of the ten patients with positive reactions. Four of these eight patients had positive serological reactions to KP1_p307 protein on the first day after hospital admission (Table 2). The other two patients (I and J) were observed to seroconvert (Figure 5) on the 5^th^ and 10^th^ day after admission, respectively, and to still have anti-KP1_p307 antibody 4 months after infection (on the 110^th^ day). These results confirm that the antibodies against the KP1_p307 protein were elicited after infections. Therefore, the KP1_p307 protein could be used to serodiagnose *K. pneumoniae* PLA.

### Diagnostic application of KP1_p307 protein in *K. pneumoniae* infections other than PLA

The diagnostic application of KP1_p307 protein in *K. pneumoniae* infections other than PLA was further examined (Table 3). Sera from 12 non-PLA patients were obtained from VGH and their *K. pneumoniae* isolates were also collected. The capsular type was determined by *wzc* genotyping and the presence of KP1_p307 was determined by PCR. The three strains with K1 or K2 capsular type all possess KP1_p307 and five out of the 9 non-K1/K2 strains (55.6%) have KP1_p307. Four of the 8 sera (50%) collected from patients infected with KP1_p307-positive *K. pneumoniae* strains when they visited the hospital were immunoreactive with the recombinant KP1_p307 protein.

**Table 3:** **The immuno-reaction to KP1_p307 protein of sera from the 12 patients with** ***K. pneumoniae*** **infections (other than PLA)**

**Patients**	**Diagnosis**	**Capsular type** ^**1**^	**KP1_p307 PCR**	**KP1_p307 immunoreaction**
1	primary bacteremia	K2	+	+
2	necrotizing fasciitis with bacteremia	K2	+	−
3	primary bacteremia	K1	+	−
4	necrotizing fasciitis	K62	+	+
5	primary bacteremia	K58	+	−
6	renal abscess with bacteremia	K5	+	+
7	primary bacteremia	K74	−	−
8	cholecystitis with bacteremia	K58	−	−
9	cholecystitis with bacteremia	K46	−	−
10	urinary tract infection	K58	+	+
11	pneumonia with bacteremia	K68	+	−
12	primary bacteremia	K28	−	−

^1^Capsular types of these strains were determined by using *wzc* genotyping.

## Discussion

In this study, we identified an immunodominant antigen in a *K. pneumoniae* PLA strain by a proteomic approach and evaluated its application to the serodiagnosis of *K. pneumoniae* PLA. The sensitivity of *K. pneumoniae* PLA detection was 71.4% (10/14) and four out of six sera (66.7%) collected on the first day of hospitalization for *K. pneumoniae* PLA were positive for anti-KP1_p307 antibodies. Our previous study indicated that 80% of patients with PLA were caused by *K. pneumoniae* infection [15]. Therefore, the case of non-*K. pneumoniae* PLA was relatively few. We included serum samples from three patients with LA not caused by *K. pneumoniae* and sera from ten healthy subjects to confirm the specificity. The results indicated that the specificity was 100% (13/13). The detection of antibodies against KP1_p307 protein could be performed directly using sera and did not require the cultivation of bacteria. Although the sensitivity and specificity of this test still need to be confirmed by using a larger number of clinical samples, we suggest that detection of anti-KP1_p307 antibody was considered to be an easy and rapid method for early screening of *K. pneumoniae* PLA. As shown in Figure 5, patient I was observed to still have anti-KP1_p307 antibody 4 months after infection (on the 110^th^ day), therefore antibody titers could persisted for months after successful treatment. If the antibodies persist, the KP1_p307 antigen could not be used in the diagnosis of *K. pneumoniae* re-infection. However, because we did not have adequate follow-up sera, how long the anti-KP1_p370 antibodies persisted could not be determined.

The KP1_p307 protein was predicted to have a signal peptide similar to that of the KP1_p307 protein present in the fraction secreted from bacteria. The amino acid sequences of KP1_p307 revealed approximately 50% identity to a hypothetical protein of *L. rossiae* and a hypothetical protein of *E. dispar*. Hence, the cross-reactivity of antibodies against *L. rossiae* and *E. dispar* to KP1_p307 protein may occur. However, the infections caused by *L. rossiae* and *E. dispar* were extremely rare. There were neither *L. rossiae* nor *E. dispar* isolates in the bacteria collection of NTUH, Taipei VGH and Chang Gung Memorial Hospital, thus we have difficulties to examine the crossreactivity. But, we expected that the possible crossreactivity would not interfere in the application of KP1_p307 to the serodiagnosis of *K. pneumoniae* PLA. Moreover, the function of KP1_p307 protein remains unknown and difficult to study. Although the presence of this protein was correlated with *K. pneumoniae* PLA, knock out of KP1_p307 did not decrease the virulence of *K. pneumoniae* NTUH-K2044 strain in mice even its *in vivo* ability to compete with wild type strain. Our preliminary result demonstrated that the anti-KP1_p307 antibodies could not block the infection of *K. pneumoniae* NTUH-K2044 strain in mice (data not shown). Further investigation of the functions of KP1_p307 protein and anti-KP1_p307 antibody is still needed.

Previous studies have demonstrated the correlation of the virulence o f *K. pneumoniae* K1 strains with the presence of aerobactin [25,26]. Our previous study also reported that p-*rmpA* gene (for regulator of mucoid phenotype A) was associated with *K. pneumoniae* PLA [27]. However, knockout of the *iuc* region (which encodes aerobactin) or p-*rmpA* did not decrease its virulence in mice [27,28]. Here, the KP1_p307 was also associated with *K. pneumoniae* PLA, but knockout of this gene had no influence on virulence. Because these genes (*iuc* region, p-*rmpA* and KP1_p307) are all located on the large plasmid and associated with bacterial virulence, we propose that other virulence factors might be present in the large plasmid of *K. pneumoniae*. These genes could serve as markers of bacterial virulence due to their co-inheritance together with virulence genes carried by this plasmid.

As shown in Table 1, the presence of KP1_p307 was also correlated with capsular type K1/K2. The *K. pneumoniae* strains with capsular types K1 and K2 are more virulent than those with other capsular types [25,29]. Therefore, the KP1_p307 can be used to detect not only *K. pneumoniae* PLA but also possibly other infections caused by capsular type K1/K2 strains. The majority of *K. pneumoniae* PLA strains were capsular type K1 and K2 [19]. But we did not obtain all the *K. pneumoniae* strains isolated from these 14 PLA patients in this study (Table 2); consequently the capsular types of these isolates were unknown. Besides PLA, the diagnostic application of KP1_p307 protein in *K. pneumoniae* other infections was also examined. All strains with K1 or K2 capsular type possess KP1_p307 gene and 55.6% of non-K1/K2 strains also have KP1_p307. But only 50% of sera collected from patients infected with KP1_p307-positive *K. pneumoniae* strains were immunoreactive with KP1_p307 protein. Because these sera samples were collected at the first day when the patients visited the hospital, the anti- KP1_p307 antibodies may not yet be elicited at this very early time point. These results demonstrated that the detection of anti-KP1_p307 antibody implicated the infections by several capsular types (particularly K1 and K2) of *K. pneumoniae* which carry a virulent plasmid.

There are several immunogenic antigens of *K. pneumoniae*, such as OmpA and OmpK36 etc., have been reported previously [30]. The immuno-reactivity to OmpK36 of sera obtained from PLA patients was also examined. But the sera samples from all the *K. pneumoniae* PLA patients were not immuno-reactive to the recombinant OmpK36 protein (data not shown). We speculated that OmpK36 antigen located on the outer membrane might be masked by capsule and might not be exposed, because the strains causing PLA were observed to be heavily encapsulated [22]. The antibodies against OmpK36 might be produced at a later stage or might not be elicited during *K. pneumoniae* PLA infections. Moreover, OmpK36 protein is commonly distributed among *K. pneumoniae* strains and has sequence similarity to that of *E. coli*. These results indicated that KP1_p307 would be a better antigen than OmpK36 to serodiagnose *K. pneumoniae* PLA.

## Conclusions

In conclusion, an immunodominant antigen, KP1_p307, was identified in a *K. pneumoniae* strain causing PLA. The KP1_p307 gene was located on the large plasmid and more prevalent in PLA strains and capsular type K1/K2 strains, but deletion of KP1_p307 did not decrease the virulence of this strain in mice. The high sensitivity and specificity demonstrated here indicates that the Kp1_p307 protein could be used to serodiagnose *K. pneumoniae* PLA or other infections.

## References

[1] Chang SC, Fang CT, Hsueh PR, Chen YC, Luh KT (2000). Klebsiella pneumoniae isolates causing liver abscess in Taiwan. Diagn Microbiol Infect Dis.

[2] Chung DR, Lee SS, Lee HR, Kim HB, Choi HJ, Eom JS, Kim JS, Choi YH, Lee JS, Chung MH, Kim YS, Lee H, Lee MS, Park CK, Korean Study Group for Liver Abscess (2007). Emerging invasive liver abscess caused by K1 serotype Klebsiella pneumoniae in Korea. J Infect.

[3] Fang FC, Sandler N, Libby SJ (2005). Liver abscess caused by magA+ Klebsiella pneumoniae in North America. J Clin Microbiol.

[4] Karama EM, Willermain F, Janssens X, Claus M, Van den Wijngaert S, Wang JT, Verougstraete C, Caspers L (2008). Endogenous endophthalmitis complicating Klebsiella pneumoniae liver abscess in Europe: case report. Int Ophthalmol.

[5] Keynan Y, Karlowsky JA, Walus T, Rubinstein E (2007). Pyogenic liver abscess caused by hypermucoviscous Klebsiella pneumoniae. Scand J Infect Dis.

[6] Kohayagawa Y, Nakao K, Ushita M, Niino N, Koshizaki M, Yamamori Y, Tokuyasu Y, Fukushima H (2009). Pyogenic liver abscess caused by Klebsiella pneumoniae genetic serotype K1 in Japan. J Infect Chemother.

[7] Liew KV, Lau TC, Ho CH, Cheng TK, Ong YS, Chia SC, Tan CC (2000). Pyogenic liver abscess–a tropical centre’s experience in management with review of current literature. Singap Med J.

[8] Lok KH, Li KF, Li KK, Szeto ML (2008). Pyogenic liver abscess: clinical profile, microbiological characteristics, and management in a Hong Kong hospital. J Microbiol Immunol Infect.

[9] Moore R, O’Shea D, Geoghegan T, Mallon PW, Sheehan G: **Community-acquired Klebsiella pneumoniae liver abscess: an emerging infection in Ireland and Europe.** *Infection* 2013, **41**(3):681-686. 10.1007/s15010-013-0408-0 23381876

[10] Nadasy KA, Domiati-Saad R, Tribble MA (2007). Invasive Klebsiella pneumoniae syndrome in North America. Clin Infect Dis.

[11] Pastagia M, Arumugam V (2008). Klebsiella pneumoniae liver abscesses in a public hospital in Queens, New York. Travel Med Infect Dis.

[12] Rahimian J, Wilson T, Oram V, Holzman RS (2004). Pyogenic liver abscess: recent trends in etiology and mortality. Clin Infect Dis.

[13] Siu LK, Yeh KM, Lin JC, Fung CP, Chang FY (2012). Klebsiella pneumoniae liver abscess: a new invasive syndrome. Lancet Infect Dis.

[14] Lin YT, Liu CJ, Chen TJ, Chen TL, Yeh YC, Wu HS, Tseng CP, Wang FD, Tzeng CH, Fung CP (2011). Pyogenic liver abscess as the initial manifestation of underlying hepatocellular carcinoma. Am J Med.

[15] Tsai FC, Huang YT, Chang LY, Wang JT (2008). Pyogenic liver abscess as endemic disease, Taiwan. Emerg Infect Dis.

[16] Lee SS, Chen YS, Tsai HC, Wann SR, Lin HH, Huang CK, Liu YC (2008). Predictors of septic metastatic infection and mortality among patients with Klebsiella pneumoniae liver abscess. Clin Infect Dis.

[17] Fang CT, Chen YC, Chang SC, Sau WY, Luh KT (2000). Klebsiella pneumoniae meningitis: timing of antimicrobial therapy and prognosis. QJM.

[18] Kurupati P, Chow C, Kumarasinghe G, Poh CL (2004). Rapid detection of Klebsiella pneumoniae from blood culture bottles by real-time PCR. J Clin Microbiol.

[19] Chuang YP, Fang CT, Lai SY, Chang SC, Wang JT (2006). Genetic determinants of capsular serotype K1 of Klebsiella pneumoniae causing primary pyogenic liver abscess. J Infect Dis.

[20] Chou HC, Lee CZ, Ma LC, Fang CT, Chang SC, Wang JT (2004). Isolation of a chromosomal region of Klebsiella pneumoniae associated with allantoin metabolism and liver infection. Infect Immun.

[21] Ma LC, Fang CT, Lee CZ, Shun CT, Wang JT (2005). Genomic heterogeneity in Klebsiella pneumoniae strains is associated with primary pyogenic liver abscess and metastatic infection. J Infect Dis.

[22] Fang CT, Chuang YP, Shun CT, Chang SC, Wang JT (2004). A novel virulence gene in Klebsiella pneumoniae strains causing primary liver abscess and septic metastatic complications. J Exp Med.

[23] Hsieh PF, Lin HH, Lin TL, Wang JT (2010). CadC regulates cad and tdc operons in response to gastrointestinal stresses and enhances intestinal colonization of Klebsiella pneumoniae. J Infect Dis.

[24] Pan YJ, Lin TL, Chen YH, Hsu CR, Hsieh PF, Wu MC, Wang JT (2013). Capsular types of Klebsiella pneumoniae revisited by wzc sequencing. PLoS One.

[25] Nassif X, Sansonetti PJ (1986). Correlation of the virulence of Klebsiella pneumoniae K1 and K2 with the presence of a plasmid encoding aerobactin. Infect Immun.

[26] Yu WL, Ko WC, Cheng KC, Lee CC, Lai CC, Chuang YC (2008). Comparison of prevalence of virulence factors for Klebsiella pneumoniae liver abscesses between isolates with capsular K1/K2 and non-K1/K2 serotypes. Diagn Microbiol Infect Dis.

[27] Hsu CR, Lin TL, Chen YC, Chou HC, Wang JT (2011). The role of Klebsiella pneumoniae rmpA in capsular polysaccharide synthesis and virulence revisited. Microbiology.

[28] Hsieh PF, Lin TL, Lee CZ, Tsai SF, Wang JT (2008). Serum-induced iron-acquisition systems and TonB contribute to virulence in Klebsiella pneumoniae causing primary pyogenic liver abscess. J Infect Dis.

[29] Yeh KM, Kurup A, Siu LK, Koh YL, Fung CP, Lin JC, Chen TL, Chang FY, Koh TH (2007). Capsular serotype K1 or K2, rather than magA and rmpA, is a major virulence determinant for Klebsiella pneumoniae liver abscess in Singapore and Taiwan. J Clin Microbiol.

[30] Kurupati P, Teh BK, Kumarasinghe G, Poh CL (2006). Identification of vaccine candidate antigens of an ESBL producing Klebsiella pneumoniae clinical strain by immunoproteome analysis. Proteomics.

